# General Nursing Council Decides against Publicity

**Published:** 1920-10-23

**Authors:** 


### NURSES AND THE HOURS OF EMPLOYMENT BILL.

### General Nursing Council Decides Against Publicity.

The second autumn meeting of the General Nursing Council was held on Friday, October 15, at the Ministry of Health. Mr. J. C. Priestley, K.C., the Chairman of the Council, was in the chair, and the following members were present: The Rev. G. B. Cronshaw, M.A., Sir Thomas Jenner Verrall, M.D., Dr. E. W. Goodall, M.D., Dr. Bedford Pierce, M.D., Lady Hobhouse, the Hon. Mrs. Eustace Hills, Miss Batty Tuke, Miss A. Cattell, Mr. T. Christian, Miss A. Coulton, Miss R. Cox-Davies, R.R.C., Miss A. Dowbiggin, C.B.E., R.R.C., Mrs. E. G. B. Fenwick, Miss A. Lloyd Still, C.B.E., R.R.C., Miss M. MacCallum, Miss Isabel Macdonald, Miss Peterkin, Miss E. Smith, Miss E. C. Swift, Miss M. E. Sparshott, C.B.E., R.R.C., Miss S. E. Villiers, Miss C. Worsley, Miss C. S. Yapp.

The minutes of the last meeting were taken as read, and the next item on the agenda, the letter from the Ministry of Labour concerning the Hours of Employment Bill (adjourned from the last meeting) was then mentioned by the Chairman. He said there had been two meetings of people who were particularly interested, such as nurses, matrons, and others, that further discussion was required, and he proposed that the matter should be adjourned to a later portion of the meeting. He further ruled that the succeeding item on the agenda, the letter from the General Nursing Council for Ireland, should be taken later after Miss MacCallum's motion. This motion, according to the agenda paper, No. 5, was as follows: "That during the discussion of the Rules, which are still *sub judice*, the Press be excluded."

### The Admission of the Press.

Miss MacCallum stated that nobody could be more anxious than she that the Press should be admitted to the meetings of the General Nursing Council, whose whole proceedings should, she thought, as far as possible be made public. At the same time owing to the action of two papers [not The Hospital] which, in reporting the meeting of September 23, published extracts from the rules, which were stated to be *sub judice*, it would in her opinion be wise for the Council to protect themselves against such a possibility on this occasion, which might also be taken as an act of discourtesy to the Minister of Health, who had not passed the rules.

The Chairman reminded the meeting of the Council's resolution of July 16 authorising the admission of the Press and the amendment of the same date giving power to any member of the Council at any time to request that the meeting should go into *camerâ*. Miss MacCallum's resolution to-day was that on the discussion of the Rules the proceedings should be held *in camerâ*. Miss Macdonald seconded the resolution, saying that premature publicity led to misapprehension on the part of nurses, and it was carried unanimously.

Miss Villiers then proposed that the Minister of Labour's letter concerning hours of employment be also taken *in camerâ*. This was seconded by Miss Cattell, but before it was put to the meeting Miss MacCallum stated that in her view the matter affected the general public a good deal, and that the public should know something of the discussion. On Dr. Goodall asking for information why it was proposed that the discussion on this subject should be private, the Chairman explained that the old Bill, which had been placed on the table of the House of Commons last session, is to be amended and a new one brought forward in substitution for it; such a new Bill has not yet been drafted, and the public discussion of a hypothetical Bill, not yet brought before the House of Commons, was to be deprecated. The members of the Council who had discussed the matter the previous day were not in possession of copies of the Bill.

Sir T. Jenner Verrall pointed out the thorniness of the question as displayed by the Chairman's explanation. He desired to have the matter thoroughly discussed and settled by those members of the Council who had it in hand, so that when the subject was brought up for discussion by the Council itself, the Council might have before it a decided and unanimous statement, and not what might be termed a minority report.

### References from Two Ministries.

The Chairman then read certain correspondence which had passed between the Minister of Labour and the Minister of Health, together with a request from the latter to the General Nursing Council asking it to proceed to consider at an early date the scheme submitted by the College of Nursing. The Council, however, need not regard this reference as limited to the College scheme, and the Minister would be glad to consider observations which it might desire to make not only in regard to the scheme itself, but generally. It would be convenient if its observations dealt separately with institutional nursing, district nursing, and private practice. The Minister of Labour had further informed the Minister of Health that copies of the draft College of Nursing Scheme had been sent to the British Hospitals Association, Queen Victoria Jubilee Nurses' Association, the National Union of Trained Nurses, the Royal British Nurses' Association, and the Nurses' Co-operation with a view to obtaining the observations of those bodies. He would be glad if it could be placed before Dr. Addison, and it would be an advantage if the views of the General Nursing Council could be obtained upon it and a copy of the scheme laid before the Council.

### The College of Nursing Scheme.

Mr. Priestley proceeded to read the College draft scheme for inclusion in a Special Order in the Hours of Employment Bill (already published in The Hospital of June 5, p. 262) as follows:

"1. That all registered nurses and other persons actually engaged in rendering services in direct connection with the nursing of the sick, excepting maternity nurses, be included in the provision of the Special Order.

"2. That for nurses in institutions for the sick, including those where probationers are in training, for nurses in district nursing institutions, and those employed by district nursing associations, and for nurses engaged in public-health work, the maximum working hours be fifty-six per week, taken over a period of four weeks, the time on duty not to exceed ten hours in twenty-four hours. Any time worked in excess of the maximum to be compensated by extra off-duty, given during the nurse's normal working hours, and if extra off-duty time due to the nurse exceeds forty-eight hours, board and residence to be provided, or the monetary equivalent, if preferred by the employer.

"3. That in institutions for trained nurses on the co-operative system, and other institutions supplying nurses for private cases and in nursing homes, the maximum working hours be fifty-six per week, taken over a period of fourteen days, any time worked in excess of this maximum to be compensated by the same number of hours given as extra off-duty during the nurse's normal working hours, and the nurses to receive, in addition, the fee due from the patient for the extra time worked by her, together with board and residence, or the monetary equivalent, if preferred by the employer."

On Miss Villiers' motion being put to the meeting, eighteen members voted for it, and it was carried. The Chairman then stated that the proceedings to follow were private, and asked if the Press present would give their word that they should be so treated. Mrs. Bedford Fenwick, however, protested against the presence of the Press, pointing out that the resolution declared that the matter should be taken *in camerâ*. The Chairman acquescing, the Press then withdrew.

At a later stage of the meeting it was resolved, on the motion of Miss Seymour Yapp, that a day should be fixed for the periodic meetings of the Council, and the second Friday in each month, at 2 p.m., was decided on. On Miss Cox Davies' motion, it was resolved that the posts of Registrar's Assistant and Registration Clerk, being open to trained women, should be advertised, and that the age limit should be fifty.

